# What protects older Romanians in Switzerland from loneliness? A life-course perspective

**DOI:** 10.1007/s10433-020-00579-2

**Published:** 2020-09-17

**Authors:** Ruxandra Oana Ciobanu, Tineke Fokkema

**Affiliations:** 1 Institute of demography and socioeconomics, Centre for the Interdisciplinary Study of Gerontology and Vulnerability and Swiss National Centre of Competence in Research LIVES, University of Geneva, Geneva, Switzerland; 2 Netherlands Interdisciplinary Demography Institute (NIDI-KNAW), University of Groningen, The Hague, The Netherlands; 3 Department of Public Administration and Sociology, Erasmus School of Social and Behavioural Sciences (ESSB), Erasmus University Rotterdam, Rotterdam, The Netherlands

**Keywords:** Older migrants, Political refugees, Loneliness, Protective factors, Coping strategies, Life course

## Abstract

The topic of loneliness among older migrants has recently gained scholarly interest. There is a particular focus on why older migrants are generally lonelier than their non-migrant peers from the destination. These studies neglect variations both within and between older migrant groups. Our qualitative study is innovative for three reasons. First, it focuses on Romanian migrants aged 65+ who fled communism and aged in place in Switzerland—an understudied population of former political refugees that experiences little or no loneliness in later years. Second, it takes a life-course approach to explore experiences of loneliness during communist Romania, in the context of migration and later in life. Third, it focuses on protective and coping factors rather than risk factors. Having been through hard times in communist Romania—marked by fear and distrust among people and estrangement from society—older Romanian migrants built strength to withstand difficult times, learned to embrace solitude, and/or to relativise current hardships, if any. Upon arrival many founded or joined an association or church, which offers the opportunity to establish a sustainable social network consisting of a large pool of Romanian non-kin with a shared past and experience of migration and integration, to counteract social losses in later life. When moments of loneliness cannot be prevented (e.g. due to death of a spouse), they try to be active to distract from loneliness or ‘simply’ accept the situation. These aspects need to be taken into account in future research and when developing loneliness interventions.

## Introduction

There is a growing interest in the topic of loneliness among older migrants. This population is often portrayed as experiencing a triple jeopardy: besides inherent age-related problems, they have the experience of migration and of belonging to a minority group, with both short-term and long-term consequences for social life and general well-being. Quantitative studies show that older migrants are lonelier than their native-born peers, partly because of general risk factors like lower socio-economic status and poorer health (de Jong Gierveld et al. 2015; Fokkema and Naderi, 2013; Victor et al. 2012; van Tilburg and Fokkema 2020). Qualitative studies explore migrant-specific factors that can increase the likelihood of feeling lonely: moving to another country with a different culture in terms of values and norms, a different language, experiencing ethnic discrimination and social isolation, missing the country of birth and those left behind, and so on (Cela and Fokkema 2017; Park et al. 2019; Torensma 2014).

Our paper is triggered by an empirical puzzle we came across in our study on older Romanian migrants in Switzerland, where study participants reported few cases of current loneliness. Previous studies predominantly focused on labour migrants and their follower spouses, while older Romanian migrants in Switzerland are former political refugees who aged in place. The circumstances prior to and after migration were very different in their case compared to other migrant groups. We therefore enquire: which coping strategies and underlying factors protect Romanian migrants in Switzerland from loneliness in later life?

We take a life-course perspective and focus on how the pre-migration context (i.e. historical and political), the experience of migration and the post-migration context shape migrants’ feelings of loneliness in old age. More specifically, we are interested in how older Romanian migrants have incorporated past experiences, including loneliness, and transformed them into positive forces and strategies to prevent or overcome late-life loneliness in the foreign country. Our paper comes to fill in three gaps in existing scholarship: the topic it addresses (focusing on protective and coping factors rather than risk factors; Ciobanu et al. 2017; de Jong Gierveld and Fokkema 2015), the approach (the life-course perspective; Jopling and Sserwanja 2016; McDonald 2011) and the study population (former political refugees who have aged in place at the destination, an understudied population; Ciobanu et al. 2017).

## Theoretical framework

There is a general consensus that loneliness is an unpleasant, subjective, personal experience caused by a discrepancy between the number and quality of the relationships one desires and those one actually has (Peplau and Perlman 1982). In general, the risk of losing important social relationships is greater at older ages, due to retirement, bereavement, decline in mobility and health, and so on (Hawkley et al. 2008). The best way to shield against these social losses, which might trigger the onset of loneliness, is to develop a ‘social convoy’, a network of close relationships (confidants, best friends) that accompany an individual across the life span (Kahn and Antonucci 1980); in the coping literature this strategy is labelled as ‘problem-focused coping’ (Lazarus and Folkman 1984). To do so successfully, de Jong Gierveld and Fokkema (2015) address the need to meet the following preconditions: to want, to know how and to be able to start and pursue actions in the direction of establishing and maintaining a broad and high-quality social convoy.

The willingness to establish and maintain a network of meaningful relationships is an ongoing, time- and energy-consuming investment (Perese and Wolf 2005). In addition, one should start as early as possible, as it is difficult, if not impossible, to start building a social convoy late in life (Schoenmakers et al. 2014). Moreover, showing sincere interest towards the other and providing room for reciprocal support are crucial to the functioning of the social convoy (Antonucci et al. 2011). In terms of knowledge, one should know the opportunities that are available to meet people and develop common interests, such as volunteering, places of worship, hobbies and sport clubs (de Jong Gierveld and Fokkema 2015). In terms of ability, besides societally induced constraints, establishing and maintaining a social network require specific personal and personality traits like social skills, self-esteem, self-management abilities, openness and no attitude of self-pity (Antonucci et al. 2014; de Jong Gierveld et al. 2018).

Migration unavoidably entails a major disruption of one’s social convoy (Park et al. 2013). Close family and friends are left behind and despite regular letters, calls or text messages, it is not easy to remain emotionally close and supportive while physically at a distance (Baykara-Krumme and Fokkema 2019). As a consequence, it is likely that migrants experience a high level of loneliness immediately after migration. Yet, those who arrived as adults, like the participants of this study, may have the time to rebuild a new social convoy to protect them from loneliness later in life. Whether they will succeed depends on the three preconditions discussed above.

Lack of success in developing and maintaining a satisfying set of relationships at the destination does not automatically lead to late-life loneliness: one can also deal with the emotions evoked by social deficits (Lazarus and Folkman 1984). This so-called emotional-focused coping is often divided into two subcategories: active emotion-focused coping that includes cognitive reappraisal (acceptance, positive reframing) of the situation, and passive or avoidant emotion-focused coping that includes efforts to avoid directly dealing with the situation (e.g. seeking distraction in activities) (Folkman and Lazarus 1985). However, some scholars (e.g. Schoenmakers 2013) doubt whether the latter is actually a way of coping because, unlike the other two, it brings about a temporary rather than a structural relief. There is a high degree of consensus that emotion-focused coping becomes more prevalent with increasing age. Within the limited time horizon, lost relationships in old age are not easily replaced by new relationships. Moreover, it is likely that older adults are better able to regulate their emotions effectively when they have been subject of adversities earlier in life. Previous exposure to hardship puts the present situation in perspective, and overcoming stressful moments in the past increases one’s capacity to deal with loss later in life (the so-called steeling effect; Rutter 1987).

Which of these coping strategies are being used by older Romanians in Switzerland to protect themselves from loneliness, and which experiences over the life course are the driving forces behind the chosen strategy, are open questions that we explore in this study.

## Research methodology

This study is part of a larger project on the integration of older Romanian migrants in Switzerland and their transnational ties to Romania. For the data collection on the topic of loneliness, we used a semi-structured questionnaire with three topics: the concept of loneliness, loneliness in the Romanian community, and individual loneliness—past, present and future.

The analysis is based on fieldwork research in the Swiss cantons of Geneva and Vaud. The fieldwork was conducted between April 2013 and January 2014. The first author conducted 21 in-depth interviews, with eighteen individual migrants and three couples of migrants, all aged 65+ . Interviews were done in Romanian, although at times some participants also spoke in French. Average duration of the interviews on the topic of loneliness was 40 min. Ten interviews were conducted in Geneva and eleven interviews in Vaud. Participants were identified through different institutions Romanians are involved in (all institutional channels accounting for eight participants), through recommendations of colleagues and friends (seven participants) or using the snowballing technique (six participants). Interviews were conducted at participants’ homes (fifteen), at the first author’s workplace (three), in cafes (two), and at a participant’s workplace (one).

In terms of sample composition (see Table 1), the interviews were conducted with eleven women, seven men and three couples, adding up to a total of 24 participants. Family interviews involved both partners simultaneously, and participants answered completing each other. Nineteen participants were married, two were divorced, and three widowed. Sixteen participants were aged between 65 and 74, and eight over 75. In terms of educational level, the majority hold college or university degrees, three completed high school, and one participant completed a vocational school. Most participants worked in jobs at their own educational level, and all reported speaking fluent French. This is a privileged and self-selected group. They were able to leave Romania, something not accessible to all, and at the same time took a risk fleeing communism. There are frequent, occasional and infrequent churchgoers. Average duration of residence in Switzerland was 34.6 years, with the longest duration being 42 years, and the majority took up Swiss citizenship. Participants do not intend to return to Romania, yet do have ties to Romania and visit.

**Table 1:** Characteristics of the participants

≠	Name	Age	Gender	Marital status	Educational level	Former economic activity	Mentioned loneliness during the interview
1	Florina	73	F	Widowed	Nursing school	Nurse	Felt lonely in the past, not lonely anymore In the context of migration, health problems and her mother’s death
2	Costin	67	M	Married	University	Teacher and artist	Felt lonely in the past, not lonely anymore In the context of communism
3	Gina	65	F	Married	Vocational school	Employed by husband’s construction company	Very lonely In the context of migration, upon arrival in Switzerland
4	Mihai	76	M	Married	University	Local administration	Not lonely
5	Barbu	72	M	Divorced	University	Psychiatrist	Lonely also at present Divorce
6	Roza	83	F	Widowed	Three-year college, social work	Informal work	Lonely in the past, not lonely now After husband’s death
7	Eugenia	75	F	Married	Technical college	Technical designer	Not lonely
	Edgar	81	M	Married	University	Low-skilled jobs	Not lonely
8	Maia	80	F	Married	High school	Secretariat and accounting	Not lonely
9	Lucian	71	M	Married	University	Entrepreneur, IT	Not lonely
10	Teodora	72	F	Married	University	Nurse	Not lonely
11	Sofia	73	F	Divorced	High school	Babysitter	Moderately lonely (discreet in her attitude) In the context of communism and of parents’ death, who also lived in Switzerland
12	Tania	70	F	Married	University	Architect	Not lonely
13	Celia	67	F	Married	High school and Art school	Secretary	Not lonely
14	Bianca	67	F	Married	University	Psychologist	Not lonely
15	Marcel	92	M	Married	University	Doctor	Felt lonely in the past, not lonely anymore In the context of communism
16	Ileana	70	F	Widowed	–	Nurse	Not lonely, nostalgic
17	Vlad	81	M	Married	University	Architect, entrepreneur (still working)	Felt lonely in the past, not lonely anymore In the context of communism
18	Carmen	65	F	Married	College for nurses	Nurse	Not lonely
	Cornel	80	M	Married	University	Graphic designer	Not lonely
19	Mioara	66	F	Married	University	Architect	Not lonely
	Marcu	72	M	Married	University	Engineer	Not lonely
20	Paula	72	F	Married	University	Engineer	Not lonely
21	Gabriel	69	M	Married	University	Engineer	Not lonely

The interviews were transcribed and translated into English by the first author, who is a native Romanian. All participants were given pseudonyms to ensure confidentiality. Next, we did a content analysis of the interviews. The analysis was prompted by the observation that the participants in our research are little if at all lonely. This does not mean that they do not know what ‘being lonely’ is; some have experienced loneliness in the past. Therefore, we first analysed the interviews through the lens of factors and strategies preventing them from loneliness, and identified three fields: family and social networks, being active and accepting one’s loneliness. Second, we looked at the sources of past loneliness, if any, and observed three common loneliness-provoking experiences throughout the life course: having lived under the communist regime, when they migrated and when they faced major life events in later years. As a final step, we linked these experiences with the protective and coping factors.

## Results

Taking a life-course approach, we structure the empirical sections according to the three common experiences throughout participants’ lives. Within each of these experiences, we first discuss the factors related to loneliness, followed by how these experiences have been transformed into positive forces and strategies to prevent or overcome late-life loneliness.

### Hard times during communism

Romania was under a totalitarian communist regime between 1947 and 1989. There were people who fled, although this was exceptionally difficult to do. In other words, this is a self-selected population in terms of individual characteristics, made up of rather daring persons. Some settled in Switzerland in the 1970s and 1980s, applied for political asylum and later aged in place (Ciobanu and Fokkema 2017). Longitudinally seen, communism was a period in the participants’ life that has had an important bearing on their experiences of loneliness—both while living under the communist regime in Romania and currently in their everyday life and strategies to cope with loneliness or the way they reflect on it.

Our findings point to a very interesting observation: some participants spoke about experiencing loneliness prior to migration under the communist regime. People felt extreme loneliness because they did not feel they belonged in the Romanian political system and because the system was trying to instil fear and distrust. They felt they could not trust anyone, not even family and friends, and there was a constant fear of the secret police (Securitate). Four former political refugees illustrate the severe loneliness they felt while living under communism in Romania and how this motivated them to migrate. Costin (M, 67, Geneva) spoke about having been investigated by the secret police, as a result of which he was stigmatised and avoided by colleagues and could not trust anyone. Similarly, Vlad (M, 81, Vaud) told that during communism social networks did not provide an anchor in society and a shield from feelings of loneliness. To some extent, Vlad proposes a re-definition of loneliness, not in terms of number and quality of ties but because of lacking identification with a social system: *One feels lonely even when one has a family. To have a feeling of solitude in relation to a social regime, with an ethic, moral and political regime. As it was my case*. Marcel (M, 92, Vaud) also experienced a situation of extreme loneliness under communism. He explained that communism estranged him from society. His reaction to the political regime was to become more reactive, isolate himself and develop a life philosophy.

In sum, communism had a negative effect on social networks by making people distrust each other and turning them against each other. This enabled politically motivated control of individuals. In this sense, it had a direct impact on people’s feelings of loneliness, as the common definition of loneliness refers to the lack of social relationships one would want to have. Communism likewise contributed to alienation on a broader social level, as people did not identify with the dominant values in society.

#### Relativisation and strong character

Having lived in communist Romania has had a non-negligible impact on how participants currently cope with feelings of loneliness. Instead of comparing themselves with others, they compared their present situation to their life in the past. Participants usually felt that any situation was better than communist times, which were marked by mistrust, stigmatisation, limited freedom of speech and lack of basic resources (e.g. limited heating and electricity, food scarcity in shops). Having gone through hardship and then escaping it, participants are aware of their good fortune. Feeling lonely or complaining about how difficult life is seems futile to them because they know what it means to actually have a hard life. An ensuing impact is that participants believe the intense suffering and hardship they endured during communism made them stronger. Hence, they are not easily demoralised by challenging situations they currently encounter. Moreover, knowing that they were true to themselves and did not become informants for the secret police or did not give into communism boosted their self-esteem. They have also learned and accepted to live a solitary life. Sofia (F, 73, Vaud) expressed it as follows: *Communism toughened me up and made me become a dignified woman that, as I said: dignity, freedom and to accept to live in solitude with vinyl records and my books.* Several other participants also refer to certain personality traits that protect them from loneliness, but do not explicitly link them to communism. As Mihai (M, 76, Geneva), who has never felt lonely, stated: *I think my psychic structure [protects me from loneliness]. I am sociable, I am outgoing, I establish connections easily, […], but maybe I inspire sympathy, maybe some people like me, but I think it depends on how we are built.* Additionally, although religion was prohibited and repressed, those who were believers told that they kept their faith and managed to practice.

### The experience of migration

Another event shared by all participants is the experience of migration that marked a clear physical rupture from the extended family. Most participants migrated alone or with their nuclear family. Those who arrived on their own applied for family reunification and managed to bring along their spouse and children. One participant brought her mother, another followed her parents, and another had his brother also in Switzerland. It was very difficult to maintain contact, given that political refugees had fled communist Romania in the 1970s and 1980s, and the totalitarian regime did not fall until 1989. Considering the significant impact of migration on a person’s social network, some participants struggled with feelings of loneliness as a result. This was the case for Gina (F, 65, Geneva), who came from a large family. Although she migrated with her husband and three young children, Gina felt very lonely upon arrival in Switzerland. Not knowing anyone else and being away from her parents and siblings was very difficult for her. While not always leading to loneliness, the migration experience was difficult for some participants. For one participant in particular, Edgar (M, 81, Geneva), being associated with a low-skilled job while unable to have his diplomas recognised, starting a life in Switzerland was extremely difficult. Retrospectively, he said he elaborated a life philosophy to help him cope.

#### Social embeddedness

By using religion as a form of resistance to communism, shortly after their migration the former political refugees founded Orthodox churches and associations that still exist today. Especially churches appear to have played a role in reducing loneliness, by being an important place to meet people and establish ties. It was like that for Gina (introduced above), who overcame her feelings of loneliness by making contact with other refugees and people attending the same church she and her family did. Having a common past both in relation to communist Romania and the experience of migration and integration in Switzerland has also led to strong friendships among older Romanians. Many of the participants referred to other former refugees who attend the same church as important persons in their social convoy. Besides its role of social platform, the church plays a role of mediator, and young and older churchgoers visit the elderly whether they are still at home or in nursing homes. This applies to people like Gina and her husband, who overcame loneliness by being active in the Romanian Neo-Protestant community. She now looks out for persons at risk in the community.

When it comes more generally to social ties, Paula (F, 72, Vaud) reminds us that social networks and support received should be reciprocal. And people can benefit from this protective environment as long as they also give back: *It is ‘donant*-*donant’ [giving*–*giving back]*—*a reciprocity. Reciprocity does not exist and these persons find themselves lonely in the end. Because people invite you a few times, but when there is no reciprocity … In the end people put them aside and these are the persons who find themselves lonely*. In other words, participants are aware that the social convoy is a long-lasting investment. Another participant, Teodora (F, 72, Geneva), also stated that while having social networks is important to protect oneself from loneliness, the quality of social ties is also essential, not only their number: *Because it is better to be alone than in bad company*.

### Major life events in later life

Fellow migrants are seen as an essential part of the social network, yet the core of the community is the family. As Cornel (M, 80, Vaud) stated: *The family, the children, I do not feel lonely as long as I am surrounded by my wife, children, and grandchildren*. Accordingly, death of family members, especially a spouse, is the most feared source of loneliness. When it happens, as it did for Ileana (F, 70, Vaud), they experience feelings of loneliness. Likewise, divorce also caused loneliness, as is the case of Barbu (M, 72, Geneva). Another life event, less dramatic but still a source of loneliness, is retirement.

#### Distraction through activities and acceptance

Getting or staying engaged in activities has helped some participants cope with loneliness in later life. This includes both social activities, such as attending church, volunteering, going on trips organised by the local town hall and attending courses, and individual activities like reading, praying, having hobbies and providing care within the family and beyond. For example, Ileana, who was recently widowed, felt lonely upon the death of her husband, with whom she had a very good relationship. To overcome this difficult moment, she found support in the local Orthodox church. Her two adult daughters live in neighbouring towns, and she recounts how she keeps busy to avoid loneliness: *In the morning, I wake up and I think ‘where am I going?’ If I do not have an appointment, I go to shops. I spend an hour in the shops, I look for one thing or another, I come home, I cook, in the afternoon I walk a bit, I do, I keep busy to do some tours … and I fill the day. If one is a bit tired, one sleeps well in the evening*. Likewise, Gina experienced a slightly difficult moment when she retired and had to rethink her life and find new activities. She said that having her adult children close-by and grandchildren to look after has helped: *I got through this moment because my daughter*-*in*-*law was on maternity leave and now I replaced her*. She does not fear being lonely in the future because she lives near her adult children. They actually bought land and built houses for their children and themselves on the same property.

One important aspect raised by several participants is the fact that even getting involved in activities has limitations. You can be busy for the time you are participating in an activity, doing a hobby or going on a trip, but, as expressed by Marcu (M, 72, Vaud): *[…] these [activities] are three, 4* h *and then it’s over*; once you come home and there is no one there, you start feeling the loneliness.

These and some other participants who could not avoid the loneliness-provoking effects of major life events in later life spoke further about accepting their feelings and situation. While this is not a strategy to buffer loneliness in itself, it is an evaluation of their life and a realisation that they have these feelings despite having tried to get over them, they are still there so they need to accept them and live with them. Barbu tells it as follows: *Since I got divorced I am alone. For almost twenty years. … I am not really alone, my son comes over to my place more often than my daughter. I go out, I have some neighbours. So, in general, one does not feel alone, but there are moments … while somebody advances in age, they [feelings of loneliness] become stronger, but generally, I got used to loneliness and this is how it is … It would be difficult to find a partner*.

Finally, religion interplays with solitude and loneliness in the context of ageing. Some participants spoke about enjoying and wanting to have moments of solitude, but not loneliness. This is linked particularly to old age, when solitude is considered to be also positive, as people appeared to need the time to reflect on their life and give it meaning. In this sense, several participants spoke about needing to be alone in order to reflect and reconnect with God. As Sofia (F, 73, Vaud) states: *I need this solitude, because in order to prepare for the great meeting [with God] one needs to experience solitude, not to be lonely, solitude.*

## Discussion and conclusions

This study focused on a group of older migrants who, in contrast to the general picture, show almost no signs of loneliness: Romanians who fled communism as adults and aged in place in Switzerland. Accordingly, instead of looking at general and migrant-specific risk factors of loneliness in old age, we explored which coping strategies they have used throughout the life course, as well as the underlying factors, that protected them against loneliness in later life. This has led to a number of interesting insights.

Firstly, although the participants do not feel lonely nowadays, some of them went through one or more episodes of loneliness in their life. Like the experiences of other migrants, the initial period after migration was a difficult and sometimes lonely one. Migration separated them from family, sometimes even from their own children for a period of time. Fortunately, they migrated mostly within the nuclear family. Yet the episode before migration—living under communism in Romania—seemed to be even more stressful and for more participants a source of loneliness. Hardship and the sociopolitical system made some feel isolated, or they felt they needed to isolate themselves in order to survive it. Participants spoke about feelings of alienation, within families—where spouses did not trust each other, adult children did not trust parents, and vice versa—and in relation to the totalitarian system. The other causes for past loneliness, not related to their migrant-specific background, were loss of a partner due to death or divorce and retirement. These findings make clear the importance of exploring older adults’ life histories of loneliness in future research. In this particular case, it shows that past loneliness is not a risk factor for experiencing loneliness later in life, and that migration is not the experience leading to the most intense feelings of loneliness.

Secondly, their negative experiences in communist Romania have had a positive impact in protecting them from current loneliness by adapting two types of coping strategies: problem-focused and active emotion-focused. With regard to the problem-focused strategy, by regaining freedom of speech and practicing religion as well as developing self-management abilities and higher self-esteem, soon after arrival in Switzerland they founded associations and Orthodox churches that still exist to this day. This enabled them to establish relationships with people with a common past and interests. Through continuous investment and reciprocity in these relationships, enduring friendships were realised. In other words, they met the preconditions: to want, to know and to be able (see theoretical framework) in order to develop and maintain a well-functioning social convoy at the destination. It is interesting to note that this also refers to the concept of ‘social resilience’ of Cacioppo et al. (2011), which is ‘the capacity to foster, engage in, and sustain positive relationships and to endure and recover from life stressors and social isolation. Its unique signature is the transformation of adversity into personal, relational, and collective growth through […] developing new relationships, with creative collective actions’ (p. 44). This also confirms the findings of Klok and colleagues (2017) that like orientation to the mainstream population, a strong sense of belonging to one’s one group can be a protective mechanism against loneliness. With regard to the active emotion-focused strategy, hard times during communism fostered a positive reframing of difficulties they have been confronted with in later years and/or acceptance of living in solitude. Hence, besides social resilience, their difficult past made them build individual resilience too. Simultaneously, some of these persons have elaborated a life philosophy, sometimes related to religion, that shields them from loneliness. In sum, these findings suggest support for the steeling effect (see theoretical framework) and highlight once again the importance of the life course perspective: because of negative experiences in the past, older Romanian migrants seem to be better equipped to deal with migration-related and later life adversities.

Thirdly, when unpleasant major life events occur later in life, emotional-focused coping strategies become prevalent, moving away from the emotions evoked by social deficits either actively (here: acceptance) or passively (here: seeking distraction in activities). This is especially the case after the death of a spouse, as this loss cannot be denied or replaced. It is worth noting that the observed shift from problem- to emotional-focused coping strategies with increasing age is consistent with previous studies (Folkman et al. 1987; Hansson et al. 1986; Thoits 1995). Also relevant is that according to the participants, keeping engaged in activities after an unpleasant major life event is only a temporary distraction from thinking about loneliness. The participants’ view supports the notion by Schoenmakers (2013) that ‘reducing the perceived importance of the social deficiency […] by being distracted from it by other activities, one can make the loneliness less prominent for a certain period of time’ (p. 15).

Fourthly, there are certain personal and personality traits protecting older Romanian migrants from loneliness, like being sociable and outgoing and the individual’s psychic structure. These are continuous throughout life, and accumulate and strengthen with time. Perhaps the most outstanding example is religiosity, a constant element in the lives of older Romanian migrants, which cuts across all the mentioned strategies to prevent or overcome loneliness through faith in God as a comrade when living in communist Romania and facing dramatic life events, by attending church (which acts as a meeting place and where one has developed and maintained a social network), and lastly by getting involved in religious activities as a way of keeping busy and distracting oneself from thoughts of loneliness (for more details, see Ciobanu and Fokkema 2017). The finding that religiosity plays such a prominent role is not surprising, as several studies show that private and public religious activities are especially beneficial for the well-being of migrants (Kim 2013; Klokgieters 2019; Roh et al. 2013).

Our study is not without limitations. The study is cross-sectional: although we asked participants about their past and present feelings of loneliness, they told their story from the perspective of the present. They might recollect past events only partially and/or give them less importance than events that occurred recently. Despite these limitations, feelings of loneliness mark one’s existence and therefore people’s recollections about them are quite accurate. Moreover, we started the interview by measuring loneliness, using the 11-item loneliness scale developed by de Jong Gierveld (de Jong Gierveld and Kamphuis 1985). This scale has been used in several surveys and has proven to be a reliable and valid instrument, yet it is not permitted for use with individual cases (de Jong Gierveld and Tilburg 1999) and has never been validated in the Romanian language; this is why loneliness scores are not shown in the table. Nevertheless, starting with the scale gave us two benefits. First, this loneliness scale does not use the word ‘loneliness’ in any of the 11 items; therefore, it does not influence participants’ responses by making them feel stigmatised for being lonely. It simultaneously allows participants to focus their attention on this phenomenon and the way they experience it, making the responses to the ensuing in-depth interview more accurate. Second, we noticed a consistency between participants’ scores on the loneliness scale and their discourse about personal loneliness during the interviews. All these strengthen our belief that results are relevant in relation to the loneliness feelings of this population. Another critique could be that we have a small number of participants. Still, by conducting in-depth interviews we gained a good grasp of past feelings of loneliness and strategies used to prevent and overcome these feelings.

Looking further, we believe that the literature on loneliness among older migrants, and also among older natives, would benefit from studies that take a life-course perspective. These studies, just like ours, are able to identify the lessons learned and tools acquired in the past that might serve to deal with present situations of loneliness. They also provide better insight into the impact of the pre-migration context of loneliness in later life, which helps avoid homogenising older migrants as a socially vulnerable group.
